# A Low-Protein Diet for Diabetic Kidney Disease: Its Effect and Molecular Mechanism, an Approach from Animal Studies

**DOI:** 10.3390/nu10050544

**Published:** 2018-04-27

**Authors:** Munehiro Kitada, Yoshio Ogura, Itaru Monno, Daisuke Koya

**Affiliations:** 1 Department of Diabetology and Endocrinology, Kanazawa Medical University, Uchinada, Ishikawa 920-0293, Japan; namusan1192@gmail.com (Y.O.); imonno@kanazawa-med.ac.jp (I.M.); koya0516@kanazawa-med.ac.jp (D.K.); 2 Division of Anticipatory Molecular Food Science and Technology, Medical Research Institute, Kanazawa Medical University, Uchinada, Ishikawa 920-0293, Japan

**Keywords:** low-protein diet, very low-protein diet, diabetic kidney disease, autophagy, mammalian target of rapamycin complex 1, malnutrition

## Abstract

A low-protein diet (LPD) can be expected to retard renal function decline in advanced stages of chronic kidney disease (CKD), including diabetic kidney disease (DKD), and is recommended in a clinical setting. Regarding the molecular mechanisms of an LPD against DKD, previous animal studies have shown that an LPD exerts reno-protection through mainly the improvement of glomerular hyperfiltration/hypertension due to the reduction of intraglomerular pressure. On the other hand, we have demonstrated that an LPD, particularly a very-LPD (VLPD), improved tubulo-interstitial damage, inflammation and fibrosis, through the restoration of autophagy via the reduction of a mammalian target of rapamycin complex 1 (mTORC1) activity in type 2 diabetes and obesity animal models. Thus, based on animal studies, a VLPD may show a more beneficial effect against advanced DKD. Previous clinical reports have also shown that a VLPD, not a moderate LPD, slows the progression of renal dysfunction in patients with chronic glomerular nephritis. However, there is insufficient clinical data regarding the beneficial effects of a VLPD against DKD. Additionally, the patients with CKD, including DKD, are a high-risk group for malnutrition, such as protein–energy wasting (PEW), sarcopenia, and frailty. Therefore, an LPD, including a VLPD, should be prescribed to patients when the benefits of an LPD outweigh the risks, upon consideration of adherence, age, and nutritional status. As the future predicts, the development of a VLPD replacement therapy without malnutrition may be expected for reno-protection against the advanced stages of DKD, through the regulation of mTORC1 activity and adequate autophagy induction. However, further studies to elucidate detailed mechanisms by which a VLPD exerts reno-protection are necessary.

## 1. Introduction

The prevalence of diabetes mellitus has been increasing worldwide in recent years. Long-term diabetes results in vascular changes and dysfunction. Complications of diabetes are the major causes of morbidity and mortality in diabetic patients. Among diabetic vascular complications, diabetic kidney disease (DKD) is recognized as both a leading cause of end-stage renal disease (ESRD) and an independent risk factor for cardiovascular diseases (CVD) [1,2]. Multifactorial management, including diet therapy, optimal glycemic control, blood pressure (BP) control using renin-angiotensin system (RAS) inhibitors, and lipid control using statin or fibrate, is recommended to suppress the progression of DKD [3,4,5,6]. Recently, novel anti-diabetic agents, including incretin-related drugs, such as a dipeptidyl peptidase-4 (DPP-4) inhibitor, a glucagon-like peptide-1 (GLP-1) receptor agonist, and a sodium glucose cotransporter 2 (SGLT2) inhibitor, showed reno-protective effects against DKD [7,8,9,10,11]. However, some patients with particularly advanced DKD rapidly progress to ESRD despite having received adequate multifactorial treatment.

Diet therapy is fundamentally important for both diabetes and DKD to maintain glucose control and suppress the progression of renal damage [12]. Regarding diet therapy, particularly in advanced renal stages, a low-protein diet (LPD) has been considered to preserve renal function in chronic kidney disease (CKD), including DKD [13,14,15,16]. However, the reno-protective effect of an LPD on DKD is controversial because previous clinical trials failed to show conclusive results. This was due to the difficulty to adhere to a daily LPD and the insufficiency of clinical data regarding the optimal amount of restricted protein intake [17,18,19,20,21]. Several previous clinical reports have shown that a very-LPD (VLPD) may provide a more beneficial effect for reno-protection than a conventional LPD, in the patients with non-DKD [22,23]. However, there are no large clinical studies showing that a VLPD has a more beneficial effect on the preservation of renal function in patients with DKD, compared to that of a conventional LPD. Additionally, the actual performance of an LPD, particularly a VLPD, in a clinical setting, has several nutritional risks, rather than benefits for reno-protection, when an appropriate diet therapy, including a sufficient energy intake, is not performed.

On the other hand, the molecular mechanisms underlying the reno-protective effect of an LPD, particularly a VLPD, against DKD have been demonstrated by numerous previous animal studies, including ours. However, its detailed mechanisms are yet to be completely elucidated. The elucidation of the mechanisms will lead to the development of a novel therapeutic option for DKD as a replacement therapy for a VLPD.

In this review, we discuss (1) the molecular mechanisms of an LPD, particularly a VLPD, and its effect against advanced diabetes-induced renal damage, based on data obtained from animal studies; (2) the current understanding of the reno-protective effect of an LPD against the progression of DKD in a clinical setting; (3) nutritional issues in patients with CKD and their relationship to an LPD; (4) expected future prospects for a novel therapy as a replacement for a VLPD.

## 2. Molecular Mechanisms by Which an LPD Exerts Reno-Protection against DKD

### 2.1. Lessons from Animal Studies

#### Protective Effects for Glomeruli

**Figure 1:**
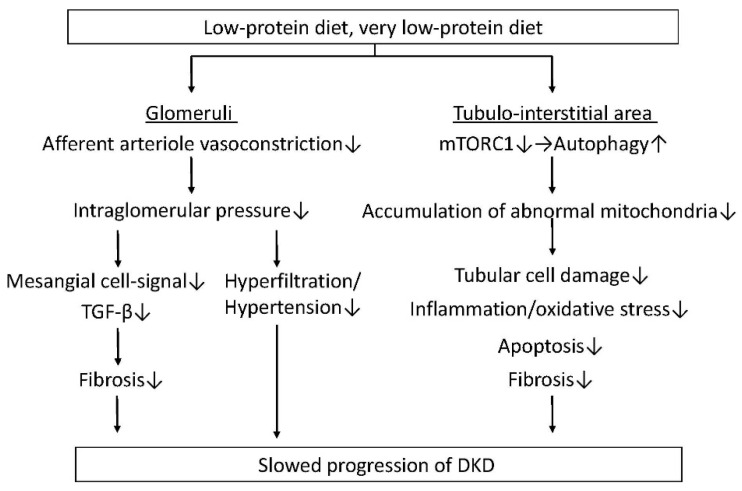
A low-protein diet (LPD) decreases intraglomerular pressure through the reduction of afferent arteriole vasoconstriction, resulting in the improvement of glomerular hyperfiltration and hypertension, and a reduction of fibrosis via growth factor-β (TGF-β) signals in mesangial cells. In addition, an LPD, particularly a very-LPD (VLPD) reduces tubular cell damage, inflammation/oxidative stress, apoptosis and fibrosis in the tubule-interstitial area by decreasing the accumulation of abnormal mitochondria, which is induced by reducing the mammalian target of rapamycin complex 1 (mTORC1) activity, and restoring autophagy. An LPD may slow the progression of diabetic kidney disease through beneficial effects in both glomeruli and the tubule-interstitial area. DKD: diabetic kidney disease.

Hyperfiltration is clinically important due to its potential to cause renal damage, which is associated with albuminuria, glomerular hypertension, and glomerulosclerosis [24]. Glimerular hyperfiltration and hypertension observed in the diabetic state are closely involved in the onset and progression of DKD [25,26,27]. The number of functioning nephrons decreases in an advanced kidney injury, which induces further glomerular hyperfiltration and hypertension in residual nephrons, accelerating the progress of renal impairment and functional deterioration. Therefore, reducing the workload of a single nephron and improving glomerular hyperfiltration and hypertension may lead to renal protection. Although RAS inhibitors have been shown to have a reno-protective effect against CKD, including DKD, in numerous basic and clinical studies [3], the mechanism by which these agents also exert a reno-protective effect through the reduction of glomerular hypertension is unclear. Recently, SGLT2 inhibitors have also been shown to have reno-protective effects, including albuminuria reduction and renal function decline, possibly through glomerular hyperfiltration via the improvement of the tubulo-glomerular feedback (TGF) system [28,29]. When focusing on dietary protein intake, a high-protein diet dilates the afferent arteriole and elevates intraglomerular pressure, leading to an increased glomerular filtration rate (GFR) [30,31]. However, glomerular hyperfiltration ultimately stimulates mesangial-cell signaling, leading to an increased transforming growth factor-β (TGF-β) release and subsequent progressive renal fibrosis and damage [32]. On the other hand, previous animal studies have shown that a low-protein intake constricts glomerular afferent arterioles and lowers intraglomerular pressure and improves glomerular hypertrophy, possibly resulting in preventing the onset and slowing the progression of DKD [30,31,33,34] (Figure 1).

## 3. Protective Effects for the Tubulo-Interstitial Area

The degree of tubulo-interstitial damage (rather than glomerular damage) is predictively related to renal function decline [35]. Therefore, the protection of renal tubular cells against diabetes-induced tubular damage leads to the preservation of renal function. However, there are few reports on whether an LPD shows a protective effect for renal tubular cells, along with glomeruli and glomerular cells, in diabetic kidneys. In addition, it was unclear whether interventional LPD treatment improves advanced diabetes-induced renal injuries, including tubulo-interstitial damage. Previously, we clearly showed that an LPD, particularly a VLPD intervention, improved advanced DKD, particularly tubulo-interstitial injuries, including fibrosis, tubular cell damage, inflammation, and apoptosis in Wistar fatty (*fa*/*fa*) rats (WFRs), which are an animal model of type 2 diabetes and obesity [36]. We also investigated the detailed mechanism by which an LPD improved advanced tubulo-interstitial damage in diabetes, focusing on autophagy and the mammalian target of a rapamycin complex 1 (mTORC1) pathway. Autophagy plays a crucial role in maintaining mitochondrial quality through the lysosomal degradation of damaged mitochondria under various stress conditions [37]. Therefore, impaired autophagy contributes to the accumulation of damaged mitochondria, resulting in increased mitochondrial oxidative stress, inflammation, and apoptosis. Although basal autophagy activity in renal tubular cells is less than that in other renal cells, such as glomerular podocytes, autophagy in renal tubular cells is induced through cellular stresses, including hypoxia and proteinuria. The activation of autophagy protects tubular cells from cellular dysfunction and apoptosis [37]. The mTORC1 pathway is recognized as an autophagy regulatory factor, and overnutrition-induced activation of the mTORC1 pathway suppresses autophagy. Since amino acids are recognized as mTORC1 activators [38], an LPD, which involves amino acid restriction, should suppress the mTORC1 pathway and induce autophagy. We demonstrated that fragmented mitochondria accumulated in diabetic proximal tubular cells (PTCs) due to an impaired autophagy, which possibly implies increased oxidative stress, inflammation and apoptosis [36] (Figure 1). An interventional LPD decreased these abnormal mitochondria in diabetic PTCs by restoring autophagy through the suppression of the mTORC1 pathway, which led to an improvement in DKD (Figure 1). In addition, a VLPD is important for revealing reno-protection because a 5.77% LPD, but not an 11.46% LPD, is needed to suppress the mTORC1 pathway and induce autophagy in the kidney [36]. Yamahara et al. also previously demonstrated that the reduction of autophagy, which is evaluated by the accumulation of p62, and the increased activity of mTORC1 were observed in renal PTCs in patients with type 2 diabetes, obesity and overt proteinuria [39]. Therefore, a VLPD may protect renal PTCs against diabetes-induced cellular damage through the reduction of mTORC1 activity and the restoration of autophagy.

Furthermore, we reported that a cyclic and intermittent VLPD (consisting of a standard diet for 3 days and a VLPD for 4 days a week), not a daily VLPD, can improve diabetes-induced renal injuries, including tubular damage, inflammation, and tubule-interstitial fibrosis, in Wistar fatty rats [40]. Therefore, a cyclic and intermittent VLPD may be a dietary regimen that is psychologically easy to continue and has less risk of malnutrition than an everyday long-term VLPD in patients with advanced diabetic nephropathy. However, a further clinical study is required to elucidate the effect of a cyclic and intermittent VLPD as a long-term treatment for advanced DKD.

## 4. Current Understanding of the Efficacy of an LPD for Reno-Protection in Patients with DKD

In the current clinical setting, the efficacy of an LPD for advanced DKD remains controversial because the evidence from clinical studies cannot sufficiently explain the reno-protective effect of an LPD. Clinical studies have not consistently shown the beneficial effects of an LPD for the preservation of renal function in DKD [17,18,19], whereas other studies have shown that an LPD has beneficial effects in slowing progressive renal function decline [20,21]. In a meta-analysis of 13 randomized control trials (RCTs) on the reno-protective effect of an LPD on DKD, Nezu et al. showed that an LPD (prescribed protein intake: 0.6–0.8/kg/day) improved the estimated glomerular filtration rate (eGFR), compared to a standard diet (prescribed protein intake: 1.0–1.6 g/kg/day) where the patients adhered to a protein-restricted diet [41]. Therefore, the difficulty of maintaining adherence to an LPD has contributed to the controversial results of previous clinical studies.

Additionally, the amount of protein restriction may be important for reno-protection. Ideura et al. previously reported that a very LPD (VLPD), consisting of less than 0.5 g/kg/day in the absence of malnutrition, significantly suppressed renal dysfunction in patients with chronic glomerular nephritis who had serum creatinine levels of more than 6.0 mg/day. However, a protein intake of more than 0.6 g/kg/day showed no beneficial effect [22]. On the other hand, the Cochrane meta-analysis based on 10 RCTs and 2000 patients with nondiabetic CKD randomized to either an LPD (0.6 g/kg/day) or a VLPD (0.3–0.6 g/kg/day) versus a standard dietary protein intake (>0.8 g/kg/day) showed a 32% reduction of the combined outcome of ESRD or mortality associated with a reduced dietary protein intake compared with a higher or unrestricted protein intake [23]. However, the optimal level of protein intake could not be confirmed. Moreover, in the Modification of Diet in Renal Disease (MDRD) Study (only a small number of DKD patients were included), the primary results were inconclusive [42]. However, several secondary analyses tended to support the conclusion that a successful protein intake restriction has a beneficial effect on the rate of renal function decline, proteinuria, and ESRD onset [43,44,45,46]. However, a long-term follow-up in this study also revealed that a higher risk of death was associated with a VLPD (0.28 g/kg/day) than with an LPD (0.58 g/kg/day) [46], which is possibly associated with malnutrition. Thus, although a VLPD without malnutrition should have beneficial reno-protective effects on CKD, including chronic glomerular nephritis, there are no clinical reports showing that a VLPD may exert a reno-protective effect against DKD. Clinical trials including RCTs for elucidating whether there is a reno-protective effect of a VLPD on DKD are difficult to perform. This is because diabetic patients with DKD are often older, and they are a high-risk group for sarcopenia or frailty.

**Figure 2:**
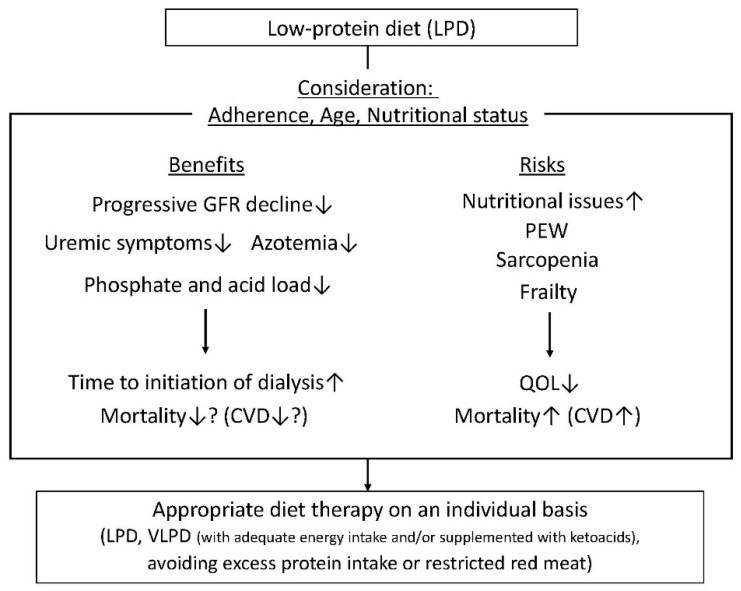
The benefits of a low-protein diet (LPD) in chronic kidney disease (CKD) patients include slowing the progression of renal function decline, a decrease in uremic symptoms and azotemia, and reductions of phosphate and acid loads, resulting in an extension of time for the initiation of dialysis and possibly reduced mortality, particularly for cardiovascular disease (CVD). The risks of an LPD include nutritional issues, including protein–energy wasting (PEW), sarcopenia and frailty. This results in a reduction of quality of life (QOL) and increased mortality due to increased CVD. Therefore, an LPD should be prescribed to patients with CKD, including diabetic kidney disease (DKD), with consideration of a patient’s adherence, age and nutritional status. Appropriate diet therapy should be evaluated on an individual basis (LPD, VLPD with adequate energy intake and/or supplemented with ketoacids, or avoidance of excess protein intake or restricted red meat).

In addition to the reduction of amino acid load, the reduction of nitrogen, phosphate and acid loads should be related to the retardation of the progression of ESRD and the initiation of dialysis through a decrease in uremic symptoms or azotemia [47,48] (Figure 2). Therefore, several guidelines regarding diet therapy in CKD, including DKD, recommend a restricted protein intake and avoiding excess protein intake (Figure 2). A comparison of the guidelines of diet therapy for DKD regarding a dietary protein intake is shown in Table 1. In a current clinical setting, a VLPD is not recommended as a diet therapy for DKD. However, a VLPD may be further considered as a clinically relevant means of suppressing renal function decline during the advanced stages of DKD, under an appropriate energy intake or supplementation of ketoacids (KAs) (as described below) (Figure 2).

## 5. Nutritional Issues in CKD and Relationship to an LPD

In patients with CKD, particularly in advanced renal stages, multiple factors, such as an accumulation of uremic toxins, hypermetabolism, inflammation, oxidative stress and insulin resistance, are involved in the decrease of skeletal muscle and fat mass, which is recognized as protein–energy wasting (PEW) [49,50]. Previous reports showed that PEW appears in approximately 20–50% of CKD patients [51,52]. Furthermore, this malnutrition occurs not only in patients with dialysis therapy but also in patients with pre-dialysis therapy. PEW is related to the enhancement of inflammation and oxidative stress, resulting in the increased morbidity of cardiovascular disease and increased mortality [50]. Sarcopenia is recognized as the decrease in skeletal muscle that is associated with ageing. In addition to ageing, chronic disease, including CKD and diabetes, is closely related to the development of sarcopenia in patients [53,54]. The patients with CKD have high morbidity from sarcopenia through mechanisms including increased myoproteolysis due to metabolic acidosis, an increase in intramuscular angiotensin II, a decrease in the number of muscle satellite (stem) cells, and an inhibition of muscle protein synthesis due to increased myostatin [53]. According to the National Health and Nutrition Survey (NHANES III), it has been reported that pre-sarcopenia was shown in half of the patients with a glomerular filtration volume <60 mL/min/1.73 m^2^ or albuminuria ≥30 mg/g Cr [55]. The frailty means that recovery from a stress factor and resistance decreases, resulting in a failure in biological functions (physical ability, mobility ability, balance ability, endurance, nutritional status, activity, cognitive function, and mood), and it is recognized as a biological syndrome that becomes weak against adverse events [56]. Frailty is extremely common among patients with CKD [57] and diabetes [54]. Bao et al. reported that of 1576 patients starting dialysis included in the final analytic cohort, 73% were frail, even among patients younger than 40 years, the prevalence of frailty was 63%. Frailty is related to increased mortality after the initiation of dialysis [58]. Thus, patients with CKD, including DKD, are basically a high-risk group for malnutrition, including PEW, sarcopenia and frailty. Therefore, when an LPD, particularly a VLPD, is considered for these patients, a sufficient energy intake is necessary to avoid PEW under appropriate diet therapy by a dedicated dietitian. There are no reports assessing the relationship of an LPD and nutritional status in patients with DKD. Noce et al. reported that in nondiabetic CKD patients, an LPD could retard renal function decline, but it worsened the nutritional state of patients [59]. Since an LPD can be expected to retard the progression of CKD, including DKD, it can be prescribed if the patient’s appetite and nutritional status are good. However, one should pay attention to patients with poor appetite, weight loss, sarcopenia or frailty, particularly in elderly people. In addition, when thinking about the cause of death in elderly patients with CKD, the frequency of renal death due to the progression of CKD is less than that of CVD-related death. Therefore, an LPD should be prescribed to patients when the benefits of an LPD outweigh the risks, upon consideration of their adherence, age and nutritional status (Figure 2).

KAs lack the amino group bound to the α-carbon of an amino acid; therefore, they can be converted to their respective amino acids without providing additional nitrogen. Previous reports have shown that a VLPD providing 0.3–0.6 g/kg/day supplemented with KA analogues of essential amino acids (EAAs) provide several potential advantages for people with advanced CKD [60,61], while maintaining good nutrition (Figure 2). However, the appropriate dose of a KA/EAA supplement has not been established. Additionally, clinical evidence based on large RCTs that examined whether a VLDP supplemented KA/EAA may exert the reno-protective effect on CKD, including DKD, is insufficient. Additional large clinical studies are necessary to elucidate these points.

## 6. Is the Source of Protein Rather Than the Amount of Protein Restriction Important for Protection against Renal Impairment?

As described above, an LPD may retard the progression of CKD toward ESRD. However, the effects of the dietary protein intake level and dietary protein food sources on the risk of ESRD in the general population remain unclear. Interestingly, Lew et al. showed that among the different food sources of protein examined, a red meat intake was strongly associated with an increased risk of ESRD in a dose-dependent manner [62]. They investigated these food sources in the Singapore Chinese Health Study, a prospective population-based cohort that recruited 63,257 Chinese adults aged 45–74 years. There were 951 cases of ESRD that occurred over a mean follow-up of 15.5 years. No association was found with the intake of poultry, fish, eggs, or dairy products, whereas soy and legumes appeared to have a slightly protective effect. In addition, substituting one serving of red meat with other sources of protein significantly reduced the risk of ESRD. Thus, avoiding red meat intake may show reno-protection in patients with CKD (Figure 2). However, further studies are necessary to investigate the underlying mechanisms of how acid load or other compounds, including the amino acids present in red meat, may aggravate the progression of CKD.

Trimethylamine-*N*-oxide (TMAO), a gut microbial-dependent metabolite of dietary choline, phosphatidylcholine (lecithin), and l-carnitine, is elevated in CKD and associated with coronary artery disease pathogenesis [63]. Red meat intake may lead to an elevated production of uremic toxins by the gut microbiota, such as trimethylamine *n*-oxide (TMAO), indoxyl sulfate, and *p*-cresyl sulfate [64]. These uremic toxins are associated with an increased risk of cardiovascular (CV) mortality [65].

## 7. Closing Remarks and Expected Future Prospects

An LPD, particularly a VLPD, can exert a reno-protective effect on CKD, including DKD, in advanced renal stages, as described above. Based on data from animal studies, the regulation of autophagy in the kidney, by the reduction of mTORC1 activity through amino acid restriction, may be one of the candidates for novel therapeutic targets against DKD [37]. However, in order to create a novel drug, as a VLPD replacement therapy, it is necessary to discover and develop the novel biomarker for the evaluation of autophagy in humans. Moreover, severe pan-amino acid restriction poses nutritional risks in patients such as PEW, sarcopenia or frailty. Therefore, avoiding malnutrition is important when performing treatment with an LPD or a VLPD. Several studies have shown that a methionine restriction (MR) extends lifespans in several species, such as rodents, fruit flies, roundworms, and yeast, possibly through an increased oxidative stress capacity and hydrogen sulfate (H_2_S) production [66,67,68,69]. Cooke et al. recently reported that an MR attenuated a kidney injury in 5/6 nephlectomy mice by down-regulating inflammation and fibrosis mechanisms [70]. Therefore, we suspect that the reno-protective effect of a VLPD on DKD in type 2 diabetes and obesity rats in our previous studies may be exerted through methionine or other amino acid restriction. However, further studies to elucidate which amino acid restriction, including methionine or an amino acid balance between essential and non-essential amino acids, are necessary to discover which has the greatest reno-protection for developing replacements for a VLPD without malnutrition for advanced stages of DKD.

## Figures and Tables

**Table 1:** Comparison of guidelines for diet therapy, particularly protein intake restriction, for diabetic kidney disease.

Guidelines	Section		Amount of Protein Intake Restriction
Standards of Medical Care in Diabetes–2018: Summary of Revisions Diabetes Care. 2018, 41 (Supple 1)	Microvascular Complications and Foot Care	2018	0.8 g/kg/day Avoiding: >20% of calories, >1.3 g/kg/day
Management of Diabetes Guideline (2016–2017)	Management for diabetic complication	2016	GFR < 30 mL/min/1.73 m^2^: 0.6–0.8 g/kg/day (GFR < 45 mL/min/1.73 m^2^: consideration of 0.6–0.8 g/kg/day) Macroalbuminuria: 0.8–1.0 g/kg/day
KDIGO 2012 Clinical Practice Guideline For the Evaluation and Management Of Chronic Kidney Disease	Management of progression and complications of CKD	2013	GFR < 30 mL/min/1.73 m^2^: 0.8 g/kg/day. Risk for progression of CKD: avoiding > 1.3 g/kg/day.
Academy of Nutrition and Dietetics/Evidence Analysis Library	Chronic Kidney Disease Evidence-Based Nutrition Practice Guideline	2011	GFR < 50 mL/min/1.73 m^2^: 0.6–0.8 g/kg/day (CKD); 0.8–0.9 g/kg/day (DKD)
The Caring for Australians With Renal Impairment Guidelines	Type 2 Diabetes: Kidney Disease	2010	No recommendation
K/DOQI Clinical Practice Guidelines and Clinical Practice Recommendation	Diabetes and Chronic Kidney Disease	2007	Stage 1–4: 0.8 g/kg/day

GFR: glomerular filtration rate; CKD: chronic kidney disease; DKD: diabetic kidney disease; KDIGO: Kidney Disease: Improving Global Outcomes; K/DOQI: Kidney Disease Outcomes Quality Initiative.
